# Hospital-based tuberculosis control activities in five cities of Latin America

**DOI:** 10.26633/RPSP.2017.95

**Published:** 2017-06-19

**Authors:** Ralfh Moreno, Rafael López, Alfonso Tenorio, Jorge Victoria, Anna Volz, Oscar Cruz, Ernesto Moreno, Carlos Quijada, Ana Hesse-de-Herrera, Sarita Aguirre, Laedi Santos, Noemi Lima, Neide Tanomaru, Antonieta Alarcon, Mirtha Del-Granado

**Affiliations:** 1 Pan American Health Organization Pan American Health Organization Washington, DC. United States of America Pan American Health Organization, Washington, DC, United States of America.; 2 Secretary of Health of Bogotá Secretary of Health of Bogotá Bogotá Colombia Secretary of Health of Bogotá, Bogotá, Colombia.; 3 Ministry of Health and Social Protection Ministry of Health and Social Protection Bogotá Colombia Ministry of Health and Social Protection, Bogotá, Colombia.; 4 Ministry of Public Health and Social Welfare Ministry of Public Health and Social Welfare Guatemala City Guatemala Ministry of Public Health and Social Welfare, Guatemala City, Guatemala.; 5 Ministry of Public Health and Social Welfare Ministry of Public Health and Social Welfare Asuncion Paraguay Ministry of Public Health and Social Welfare, Asuncion, Paraguay.; 6 Center for Epidemiological Surveillance Center for Epidemiological Surveillance São Paulo Brazil Center for Epidemiological Surveillance, São Paulo, Brazil.; 7 Health Secretary of Guarulhos Health Secretary of Guarulhos Guarulhos Brazil Health Secretary of Guarulhos, Guarulhos, Brazil.; 8 Ministry of Health Ministry of Health Lima Peru Ministry of Health, Lima, Peru.

**Keywords:** Tuberculosis, hospital services, cities, Brazil, Colombia, Guatemala, Paraguay, Peru, Latin America, Tuberculosis, servicios hospitalarios, ciudades, Brasil, Colombia, Guatemala, Paraguay, Perú, América Latina, Tuberculos, serviços hospitalares, cidades, Brasil, Colombia, Guatemala, Paraguay, Perú, América Latina

## Abstract

**Objective.:**

*To generate actionable insights for improving TB control in urban areas by describing the tuberculosis (TB) control activities of hospitals in five cities in Latin America*.

**Methods.:**

*A descriptive study of hospital-based TB control activities was conducted in 2013–2015 using a cross-sectional survey designed by the Pan American Health Organization and administered in Guatemala City, Guatemala; Guarulhos, Brazil; Bogotá, Colombia; Lima, Peru; and Asunción, Paraguay. Data were analyzed using Chi-squared, Fisher exact tests, and the Mantel–Haenszel test for Risk Ratios, as necessary (P < 0.05)*.

**Results.:**

*While variation among cities existed, most hospitals (91.3%) conducted acid-fast bacilli smears for TB diagnosis and had a quality control process (94.0%), followed national TB guidelines (95%), and reported TB cases to the respective health authorities (96%). Additionally, TB treatment was offered free of charge almost universally (97.1%). However, only 74.2% of hospitals were supervised by the national or local TB programs; 52.8% followed up on the outcome of referrals; and 39.1% offered full ambulatory TB treatment, with 68.7% using Directly-Observed Therapy*.

**Conclusion.:**

*The study underscored strengths and weaknesses in specific areas for TB control activities in hospitals and highlighted the importance and complexity of coordinating efforts among private and public hospitals and the various stakeholders. Local TB programs and health authorities should use these results to enhance the quality of TB-related actions in hospitals in similar settings*.

## RESUMEN

**Objetivo.:**

Generar información utilizable para mejorar el control de la tuberculosis en las zonas urbanas describiendo las actividades hospitalarias de control de la tuberculosis de cinco ciudades de América Latina.

**Métodos.:**

Se realizó un estudio descriptivo de las actividades hospitalarias de control de la tuberculosis mediante una encuesta transversal formulada por la Organización Panamericana de la Salud y administrada entre el 2013 y el 2015 en Ciudad de Guatemala (Guatemala), Guarulhos (Brasil), Bogotá (Colombia), Lima (Perú) y Asunción (Paraguay). Los datos fueron analizados con la prueba de la ji al cuadrado, la prueba exacta de Fisher y la prueba de asociación de Mantel-Haenszel de las razones de riesgos, según fuera necesario (P < 0,05).

**Resultados.:**

Pese a la variación observada entre las ciudades, la mayor parte de los hospitales (91,3 %) realizan frotis de bacilos acidorresistentes para diagnosticar la tuberculosis y disponen de un proceso de control de la calidad (94,0 %), siguen las directrices nacionales respecto de la tuberculosis (95 %) y notifican los casos a las autoridades de salud respectivas (96 %). Además, casi todos ofrecen tratamiento antituberculoso gratuito (97,1 %). Sin embargo, solo el 74,2 % de los hospitales está supervisado por el programa nacional o local contra la tuberculosis; el 52,8 % hace el seguimiento de la evolución de los pacientes derivados; y el 39,1 % ofrece tratamiento antituberculoso plenamente ambulatorio a los pacientes, del cual el 68,7 % corresponde al tratamiento bajo observación directa.

**Conclusiones.:**

En el estudio se ponen de relieve las fortalezas y las debilidades de aspectos específicos de las actividades hospitalarias de control de la tuberculosis, así como la importancia y la complejidad que reviste coordinar los esfuerzos entre los hospitales privados y públicos y los diversos interesados directos. Los programas locales contra la tuberculosis y las autoridades de salud deben aprovechar estos resultados para mejorar la calidad de las actividades hospitalarias relacionadas con la tuberculosis en entornos similares.

**Objetivo.:**

Originar insights proativos para melhorar o controle da tuberculose (TB) em áreas urbanas descrevendo as atividades de controle da TB em hospitais em cinco cidades na América Latina.

**Métodos.:**

Estudo descritivo das atividades de controle da TB em hospitais realizado em 2013–2015 com base em pesquisa transversal concebida pela Organização Pan-Americana da Saúde e conduzida na Cidade da Guatemala (Guatemala), Guarulhos (Brasil), Bogotá (Colômbia), Lima (Peru) e Assunção (Paraguai). Os dados foram analisados com o uso do teste qui-quadrado, teste exato de Fisher e teste de Mantel–Haenszel para razões de risco, conforme necessário (P < 0,05).

**Resultados.:**

Apesar de ter existido variação entre as cidades, a maioria dos hospitais (91,3%) realizou o teste de esfregaço de bacilos acidorresistentes para o diagnóstico de TB e dispunha de um processo de controle de qualidade (94,0%), seguiu os protocolos nacionais de TB (95%) e notificou casos de TB aos órgãos sanitários competentes (96%). Além disso, o tratamento de TB foi proporcionado gratuitamente quase como um todo (97,1%). Porém, somente 74,2% dos hospitais receberam supervisão dos programas locais ou nacionais de combate à TB; 52,8% acompanharam os desfechos dos encaminhamentos e 39,1% ofereceram tratamento de TB ambulatorial completo, sendo que 68,7% usaram o tratamento diretamente observado.

**Conclusões.:**

O estudo destacou os pontos fortes e os pontos fracos em áreas específicas das atividades de controle da TB em hospitais e ressaltou a importância e a complexidade de coordenar esforços entre hospitais públicos e privados e as diversas partes envolvidas. Os programas locais de combate à TB e as autoridades sanitárias devem se basear nestes resultados para melhorar a qualidade das ações relacionadas à TB nos hospitais em condições semelhantes.

Despite the remarkable health progress achieved by the Millennium Development Goals (1) at the global and regional levels, there remain substantial challenges for the prevention of death and disability due to communicable diseases (1). As recently as 2014, an estimated 9.6 million people developed active tuberculosis (TB) and 1.5 million died of the disease (2).

Particularly, in Latin America and the Caribbean, TB exhibits considerable variation. In 2013, for instance, TB incidence and mortality in Central America were 28 and 1.9 per 100 000, respectively; whereas in the Caribbean, these figures were as high as 70 and 8.3 per 100 000 (3), with some countries well above these averages (4). These areas of high burden represent a strategic priority in the TB elimination effort.

Beyond regional variations, TB is also influenced by urbanization. Cities tend to have a higher TB burden, especially in deprived areas where poverty, overcrowding, and poor sanitation are common (5). At the same time, a larger concentration of high-risk populations—the homeless, immigrants from high-incidence areas, drug users—further compounds the problem (6, 7).

Against this backdrop, the Pan American Health Organization (PAHO), with the support of the United States Agency for International Development (USAID) and local TB programs, developed an initiative to improve TB control in urban areas in the Region of the Americas. The “Framework for TB Control in Large Cities of Latin America and the Caribbean” (FTCLAC; 8) provides a comprehensive and multi-sectorial approach to TB control in urban areas, incorporating multiple actors and addressing key social determinants that perpetuate the disease, while taking into account the local health system structure (8).

Given the scarce information available on the practices of urban hospitals regarding patients with TB, a key component of the framework was to conduct a survey to address the gap. To this end, this study presents the findings from the FTCLAC hospital survey, with the aim of generating actionable insights that can be used to improve TB control activities at the hospital level across cities in Latin America.

## MATERIALS AND METHODS

A descriptive study with analytical components was conducted on the basis of a multi-city, cross-sectional hospital survey carried out in 2013–2015. A hospital was defined as a health care institution with in-patient care (9).

### Study instrument

The survey was developed by PAHO and consisted of three main sections: hospital identification, hospital characteristics, and activities related to TB control, for a total of 65 questions. In each institution, the tool was administered by up to two trained pollsters who followed a guidance document to ensure standardization. A pilot survey had been carried out at the San José del Callao Hospital, in Callao, Peru, in 2013. The survey was applied in hospitals of selected cities that were willing to participate and share results: Asunción (Paraguay), Bogotá (Colombia), Guarulhos (Brazil), Guatemala City (Guatemala), and Lima (Peru).

### Study sample

The sampling was purposive, aiming to use a total population technique based on information provided by local health authorities. Importantly, the selection criteria for hospitals in each city varied according to the local context. In Guatemala City, the survey was administered in hospitals belonging to a “municipal health district” that included the neighboring area of Mixco; only hospitals of median or higher complexity (HMHC), defined as those having other types of medical services beyond general medicine, were surveyed in this city. In metropolitan Lima, only public, HMHC were covered. In Asunción, the target was the metropolitan area, with only public institutions participating. In Bogotá, public and private HMHC were surveyed. Lastly, in Guarulhos all hospitals were included.

The survey was conducted with the consent of national, local, and hospital authorities. Data was collected through interviews with administrative and clinical personnel at each participating hospital. Since the survey gathered aggregated information at the hospital level and no individual patient data whatsoever, ethical approval was not required.

### Data analysis

Survey data was compiled and analyzed by PAHO. Descriptive statistics with some analytical components were conducted, and observations with missing data for the most relevant questions were not included in the analysis. Also, given that information from private hospitals was not available in some cities, the data was disaggregated by public/private hospitals to allow for more accurate comparisons across cities. When assessing statistical differences between categorical variables, a Chi-squared or Fisher exact test was used. The Mantel–Haenszel test for Risk Ratios (RR) was used to assess the magnitude of association and adjust for confounders, when necessary. *P*-values < 0.05 were considered statistically significant. The information was analyzed using Stata^®^ 13.1 (StataCorp LP, College Station, Texas, United States).

## RESULTS

Initially, 161 hospitals were invited to participate. All selected cities had a 100% response rate, except Guatemala City with 66%. Consequently, only 145 hospitals were surveyed. Moreover, given the volume of data missing from some hospitals surveyed in Guatemala City, two public and nine private hospitals were removed from the analysis. Likewise, two health care institutions in Bogotá and four in Asunción were not analyzed because they did not meet the inclusion criteria. Thus, a total of 128 institutions were included in the final analysis (Table 1). Hospitals were classified by size (based on the number of beds using terciles as cutoff points), public or private, and general or specialized. For Asunción and Lima, no data was collected from private hospitals.

Across all cities, most public hospitals had a TB program, although in Guarulhos the number was low, just 20.0% (Table 2). With respect to care standards for people with active TB (PWAT), most hospitals used guidelines that followed national recommendations (96.0%). Notably, public hospitals in Guarulhos and private hospitals in Guatemala City were not subject to supervision by the respective TB program. With regard to TB contact tracing (TBCT), private institutions in Bogotá and public hospitals in Guarulhos did not conduct this activity (Table 2). Notably, there was a statistically significant correlation between hospital size and TBCT, with larger institutions being less likely to carry out tracing (*P* < 0.05). Lastly, most hospitals notified the national or local TB program once a patient was diagnosed with TB, with an overall average of 94.5%.

**TABLE 1.** Characteristics of hospitals selected for a study on tuberculosis (TB) control activities in five cities in Latin America, 2013–2015

Characteristics		Guatemala City		Bogotá		Guarulhos		Lima		Asunción		Total	
		n	%	n	%	n	%	n	%	n	%	n	%
Financing source	Public	10	47.6	20	29.4	5	38.5	17	100.0	16	100.0	68	53.1
	Private	11	52.4	41	68.3	8	61.5	0	0.0	0	0.0	60	46.9
Hospital type	General	7	33.3	49	80.3	10	76.9	11	64.7	12	75.0	89	69.5
	Specialized	14	66.7	12	19.7	3	23.1	6	35.3	4	25.0	39	30.5
Size	Small (< 70 beds)	13	61.9	15	24.6	3	23.1	2	11.8	8	50.0	41	32.0
	Medium (70–195 beds)	4	19.1	21	34.4	7	53.9	4	23.5	2	12.5	38	29.7
	Large (≥195 beds)	4	19.1	21	34.4	3	23.1	8	47.1	3	18.8	39	30.5
	No data	0	0.0	4	6.6	0	0.0	3	17.7	3	18.7	10	7.8
Total		21	100.0	61	100.0	13	100.0	17	100.0	16	100.0	128	100.0

***Source:***Prepared by the authors based on the study data.

Overall, most hospitals conducted acid-fast bacilli smears (AFB), with an average of 91.3% (Table 3). In addition, among the hospitals that performed AFB, 94.0% conducted a quality control test. With respect to TB culture and drug susceptibility testing (DST), 44.4% and 6.8% of hospitals offered these, respectively; the Xpert^®^MTB/RIF assay (Xpert), which not only diagnoses TB, but also tests for resistance to Rifampicin, was included in the latter category. Hospitals in Guarulhos and public hospitals in Bogotá did not conduct DST. Of note, there was no available information on cultures and DST for private hospitals in Guatemala City.

**TABLE 2:** Organization, guidelines, and supervision of tuberculosis (TB) activities in selected hospitals in five cities in Latin America, 2013–2015

Variable	Guatemala City				Bogotá				Guarulhos				Lima		Asunción		Total	
	Public		Private		Public		Private		Public		Private		Public		Public			
	n	%	n	%	n	%	n	%	n	%	n	%	n	%	n	%	n	%
Number of hospitals	10	100.0	11	100.0	20	100.0	41	100.0	5	100.0	8	100.0	17	100.0	16	100.0	128	100.0
Presence of TB program	8	80.0	1^a^	9.1	20	100.0	40	97.6	1	20.0	2	25.0	16	94.1	14	87.5	102	79.7
Use of national TB diagnosis and treatment guidelines	10	100.0	4^a^, ^b^	57.1	20	100.0	40	97.6	4	80.0	8	100.0	17	100.0	16	100.0	119	96.0
Hospital supervision by the national/local TB program	6	60.0	0^a^	0.0	19	95.0	37	90.2	0	0.0	3	37.5	16	94.1	14	87.5	95	74.2
TB contact tracing	4	40.0	3^c^	33.3	8	40.0	0^a^	0.0	0	0.0	2^c^	28.6	10	58.8	15	93.7	42	33.6
Notification of TB cases to national/local TB program	9	90.9	7	63.6	20	100.0	41	100.0	5	100.0	8	100.0	16	94.1	15	93.7	121	94.5

***Source:***Prepared by the authors based on the study data.

a *P*< 0.05 when compared to the public sector of the same city.

b Information available for 7 hospitals.

c Information for private Guatemala City and Guarulhos available for 9 and 7 hospitals, respectively.

In relation to TB diagnosis, most hospitals used bacteriological tests (Table 3), with an overall average of 97%. In general terms, hospitals in Bogotá and Lima always confirmed TB diagnosis using a combination of symptoms, bacteriology, and chest radiography.

Once a patient was diagnosed with TB, the case management varied significantly across cities. In Asunción, for example, 87.5% of hospitals started and continued treating patients on an ambulatory basis, whereas in Guarulhos neither public nor private hospitals did so (Table 4). When hospitals offered ambulatory TB treatment, on average 68.7% of them conducted Directly-Observed Therapy (DOT) (1st and 2nd phase). Notably, TB drugs were offered free of charge almost universally (97.1%), the exception being the private sector in Guatemala City (only 33.3%).

Besides providing ambulatory TB treatment, most hospitals (84.4%) also referred PWAT to other institutions to continue therapy (Table 4). However, confirmation of successful referral only took place in 20.0% – 67.7% of hospitals, for a total average of 52.8%. Likewise, most hospitals (87.5%) offered TB inpatient care depending on the patient’s clinical condition. Regarding multidrug-resistant TB (MDR-TB), 95.8% of all hospitals requested DST when drug resistance was suspected.

It was found that, on average, 69.5% of hospitals treated people living with HIV (PLHIV) and co-infected with TB (Table 4). In these hospitals, TB screening in PLHIV was nearly universal and usually based on symptoms (99%); the percentage of patients initially diagnosed with active TB and then screened for HIV was also high, at 95%. In addition, the provision of Isoniazid preventive therapy (IPT) in PLHIV without active TB was found to be low across cities, with an average of 41.4%. Lima was a notable exception at 78.6%. For PLHIV with active TB, the provision of antiretroviral therapy (ART), overall, was 55.1%. Guarulhos had a figure of 0 since that city’s ambulatory ART was not provided in hospitals, but rather in HIV-specialized clinics.

**TABLE 3.** Availability of bacteriological tests and criteria for diagnosing tuberculosis (TB) in selected hospitals in five cities in Latin America, 2013–2015

Variable	Guatemala City				Bogotá				Guarulhos				Lima		Asunción		Total	
	Public		Private		Public		Private		Public		Private		Public		Public			
	n	%	n	%	n	%	n	%	n	%	n	%	n	%	n	%	n	%
Number of hospitals	10	100.0	11	100.0	20	100.0	41	100.0	5	100.0	8	100.0	17	100.0	16	100.0	128	100.0
Availability of acid-fast bacilli test (AFB)	9	90.0	7^a^	70.0	19	95.0	39	95.1	4	80.0	6	75.0	17	100.0	15	39.7	116	91.3
AFB quality control process	9	100.0	6	85.7	19	100.0	38	97.4	4	100.0	5	85.3	14	82.3	14	93.3	109	94.0
Availability of TB culture	3	30.0	—	—	12	60.0	20	48.8	3	60.0	2	25.0	9	52.9	3	18.7	52	44.4
Availability of drug susceptibility tests, including Xpert	3	30.0	—	—	0	0.0	3	7.3	0	0.0	0	0.0	1	5.9	1	6.2	8	6.8
Diagnosis based on:																		
Symptoms	8	80.0	7	63.6	20	100.0	41	100.0	5	100.0	7	87.5	17	100.0	10	62.5	115	89.8
Bacteriology^b^	10	100.0	10	90.9	20	100.0	41	100.0	5	100.0	6	75.0	17	100.0	15	93.7	124	97.0
Chest X-ray	7	70.0	8	72.7	20	100.0	40	97.5	4	80.0	7	87.5	17	100.0	9	56.2	112	87.5
Other tests^c^	4	40.0	1	9.1	3	15.0	12	29.3	0	0.0	3	37.5	4	23.5	1	6.2	28	21.9

***Source:***Prepared by the authors based on the study data.

a Information available for 10 hospitals.

b Bacteriology includes acid-fast bacilli test, culture, and Xpert.

c Includes biopsy, polymerase chain reaction (PCR), purified protein derivative (PPD), and computed tomography (CT scan).

In relation to TB infection control measures in health care settings, 90.5% of hospitals had an infection control committee, although Asunción had a low number, at 56.2% (Table 5). In addition, on average, only 34.1% of hospitals separated presumptive PWAT while in waiting rooms. Furthermore, only an average of 43.7% of hospitals conducted TB screening for clinical staff. Of note, public hospitals in Guatemala City, Guarulhos, and Lima had a high proportion of clinical staff with active TB. However, this difference was only statistically significant for Lima after comparing public hospitals among each other and either adjusting for hospital size (RR 2.54; 95% confidence interval [95%CI] = 1.57–4.12) or the presence of TB screening (RR 3.17; 95% CI = 1.72–5.82). In addition, despite the importance of proper ventilation for preventing TB transmission in health care settings, most hospitals did not provide enough, accurate information on this topic. Therefore, it was not possible to analyze nor include this variable in the study.

## DISCUSSION

Despite the heterogeneous sample, this study shed light on a subject for which there is little information in the Region. Given the large amount of information obtained in the results, only the most relevant outcomes were discussed.

Overall, most hospitals in the study used bacteriology coupled with symptoms as the main tools to diagnose TB—in line with current standards (10). In addition, AFBs usually underwent a quality control process. This is of particular importance given that high quality-assured bacteriology is a priority in TB control (11). Another trend across hospitals was the scarce verification of successful referral of PWAT. This is of particular concern since some lost-to-follow-up patients will not receive early treatment or any treatment at all, making clinical outcomes poorer and perpetuating the transmission of both TB and MDR-TB (12). In addition, hospitals rarely carried out TBCT. As explained in the results section, this is probably related to the institution’s size, which in turn is a proxy of a hospital’s complexity. Frequently, more complex health care institutions, such as those in Bogotá, Lima, and Guatemala City leave TBCT to public health authorities and/or to smaller, more primary health care focused hospitals like those in Asunción. Despite this, it is imperative that hospitals not offering TBCT do their utmost to ensure that patients are directed to facilities capable of comprehensive TB management and TBCT.

Regarding city trends, in Guatemala City a low proportion of both public and private hospitals conducted DOT. Moreover, several hospitals in the private sector were not willing to disclose information, and some charged for TB medication. Against this backdrop, closer surveillance of hospitals by the Guatemalan TB program and greater private hospital engagement are relevant factors to tackling TB transmission. With respect to Bogotá, the implementation of DOT in the private sector was considerably below the goal of 100% coverage, leaving room for improvement. It has to be clarified that many hospitals in Bogotá do not conduct TBCT, as this is a duty assigned to local public health authorities (13). Meanwhile, the high levels of ambulatory TB treatment and TBCT in Asunción are likely due to the public nature of the surveyed institutions, coupled with the fact that many of the hospitals were general and possibly primary health care oriented, as previously discussed.

In addition, it was found that health care workers in Lima had a higher risk of TB infection compared to other cities, a finding that is supported by other reports (14, 15). This may be partially due to the high local TB burden, among the highest in the Region (3).

In this study, many hospitals conducted AFB and diagnosed PWAT, but only a smaller proportion provided ambulatory TB treatment since most hospitals referred patients to other institutions. This appeared to be the case, particularly in Brazil, where on many occasions hospitals are the primary place in which TB is detected, but treatment is provided in primary health care facilities (16, 17). Despite this, public hospitals in Guarulhos and private ones in Guatemala City were not subject to supervision by the local TB program; were they, oversight could contribute to improvements in the quality of TB diagnosis and completion of referrals.

**TABLE 4:** Hospital management of patients with tuberculosis (TB) among selected hospitals in five cities in Latin America, 2013–2015

Variable	Guatemala City				Bogotá				Guarulhos				Lima		Asunción		Total	
	Public		Private		Public		Private		Public		Private		Public		Public			
	n	%	n	%	n	%	n	%	n	%	n	%	n	%	n	%	n	%
Number of hospitals	10	100.0	11	100.0	20	100.0	41	100.0	5	100.0	8	100.0	17	100.0	16	100.0	128	100.0
Hospitals with full ambulatory TB treatment^a^	4	40.0	2	18.2	11	55.0	10^b^	24.4	0	0.0	0	0.0	9	52.9	14	87.5	50	39.1
Hospitals implementing both phases of directly-observed therapy	1	25.0	1	50.0	8^c^	80.0	5	50.0	NA^d^	NA^d^	NA^d^	NA^d^	7	77.8	11^e^	84.6	33	68.7
Free TB medication	10	100.0	1^b,f^	33.3	20	100.0	41	100.0	NA^d^	NA^d^	NA^d^	NA^d^	17	100.0	13^g^	92.9	102	97.1
Hospital refers patients to other facility for continuous treatment^a^	7	70.0	11	100.0	19	95.0	41	100.0	5	100.0	4	50.0	15	88.2	6	37.5	108	84.4
Verification of successful referral	4	57.1	5	45.4	11	57.9	24^h^	60.0	1	20.0	1	25.0	7	46.7	4	67.7	57	52.8
Hospitalization if necessary^a^	6	60.0	4	36.4	19	95.0	40	97.6	5	100.0	6	75.0	16	94.1	16	100.0	112	87.5
Hospital requests drug susceptibility test for patients suspected of having multidrug-resistant TB	7^i^	87.5	4^i^	100.0	18^i^	100.0	34^i^	100.0	—	—	—	—	15	88.2	13^i^	92.9	91	95.8
Hospital treats HIV/TB coinfected patients	8	80.0	2^b^	18.2	14	70.0	27	65.9	5	100.0	5	62.5	14	82.4	14	87.5	89	69.5
Provision of Isoniazid preventive therapy in people living with HIV (PLHIV) without active TB	4	50.0	0	0.0	6	42.8	10	37.0	1^j^	33.3	0	0.0	11	78.6	4	28.6	36	41.4
Provision of antiretroviral therapy in PLHIV with active TB	5	62.5	1	50.0	7	50.0	19	70.4	0	0.0	0	0.0	12	85.7	5	35.7	49	55.1

***Source:***Prepared by the authors based on the study data.

a These activities can overlap.

b *P*< 0.05 when compared to the public sector of the same city.

c The public sector in Bogotáhad available information for 10 hospitals.

d Not applicable

e The public sector in Asunción had available information for 13 hospitals.

f The private sector of Guatemala City had available information for 3 hospitals.

g Asunción had available information for 14 hospitals.

h Information available for 40 hospitals.

I Information for public and private Guatemala, public and private Bogotáand Asunción available for 8, 4, 18, 34, and 14 hospitals, respectively

j Available information for 3 hospitals.

In addition, one of the key targets in TB control is the adequate management of TB/HIV (11). Surprisingly, IPT, which decreases the incidence of active TB in PLHIV (18, 19), was rarely implemented in most of the hospitals included in the study. Therefore, revision of institutional policies should be conducted to include TB prophylaxis as a regular practice in PLHIV without active TB. At the same time, it is worth noting that hospitals in our study reported higher ART coverage than that described by Regional reports (3). This could be due to either higher ART availability in urban areas compared to national averages, or to a higher provision of treatment in hospitals that care for TB/HIV coinfected patients.

Another important aspect of TB control is preventing its nosocomial transmission (20). It was found that relatively inexpensive TB infection control measures—use of surgical mask by patients, separation of presumptive PWAT in waiting rooms (20)—were not universally implemented. Additionally, most of the city hospitals conducted limited TB screening among their staff, despite that working in a health care facility is a risk factor for developing TB and MDR-TB (21). Consequently, stricter TB infection control measures, in coordination with hospital infection control committees, should be put in place.

### Limitations

This study had limitations such as representativeness—the outcomes for Bogotá, Guatemala City, and Lima reflect only the actions taken by hospitals of higher complexity, whereas in Asunción and Guarulhos all complexity levels were surveyed. In addition, the results from Guatemala City, especially those from the private sector, are likely not representative given the number of excluded hospitals. This limitation was secondary to (i) their reluctance to participate in the survey and (ii) the large quantity of missing information for those that participated. Furthermore, the unwillingness of the private sector to disclose information was noteworthy.

### Conclusion

This study found that hospitals in the surveyed cities are implementing, to various degrees, TB control activities. Many of these activities, however, need to be completed and/or enhanced, including supervision and standardization of TB diagnosis and treatment, TBCT, TB prophylaxis in PLHIV, and TB infection control measures at the institutional level. These results underscore the need to foster partnerships between the private and public sectors, as well as to coordinate among stakeholders and levels of care, especially with primary health care services. Local TB programs and health authorities may use these results to improve the quality of TB-related activities in the selected cities and in other similar settings in Latin America.

**TABLE 5.** Preventive measures and detection of tuberculosis (TB) transmission in hospital settings in five cities in Latin America, 2013–2015

Variable	Guatemala City				Bogotá				Guarulhos				Lima		Asunción		Total	
	Public		Private		Public		Private		Public		Private		Public		Public			
	n	%	n	%	n	%	n	%	n	%	n	%	n	%	n	%	n	%
Number of hospitals	10	100.0	11	100.0	20	100.0	41	100.0	5	100.0	8	100.0	17	100.0	16	100.0	128	100.0
Hospital has an infection control committee	9	90.0	7^a^	70.0	20	100.0	40	97.6	5	100.0	8	100.0	17	100.0	9	56.2	115	90.5
Includes TB control activities	8	88.9	4	57.1	16	80.0	34	85.0	4	80.0	7	87.5	12	70.6	6	66.6	91	79.3
Separation of presumptive people with active TB in waiting rooms	4	40.0	5^b^	55.5	1	5.0	16^b^	40.0	1	20.0	3	37.5	6	35.3	6^b^	42.9	42	34.1
Patients use surgical masks	9^c^	100.0	6^c^	85.7	9^d^	45.0	31	75.6	5	100.0	8	100.0	17	100.0	13	81.2	98	79.7
Staff uses N95 masks	8^e^	88.9	4^e^	66.7	18	90.0	41	100.0	5	100.0	8	100.0	15	88.2	6^e^	42.9	105	87.5
Hospital staff screened for TB	2	20.0	7^d^	63.6	6	30.0	16	39.0	2	40.0	2	25.0	13	76.5	8	50.0	56	43.7
Hospital had cases of clinical staff with TB during the last year	4	40.0	1	9.1	5^f^	26.3	10	24.4	2	40.0	0^f^	0.0	13^f^ ^,^ ^g^	81.2	1	6.2	36	29.0

a Available information for 10 hospitals.

b Information for private Guatemala City, private Bogotá and Asunción available for 9, 40, and 14 hospitals, respectively.

c Information for public and private Guatemala City available for 9 and 7 hospitals, respectively.

d *P* < 0.05 when compared to public sector of the same city.

e Information for public and private Guatemala City, and for Asunción available for 9, 6, and 14 hospitals, respectively

f Information for public Bogotá, private Guarulhos and Lima available for 19, 6, and 16 hospitals, respectively.

g *P* < 0.05 when compared to other public hospitals in other cities and after adjusting for possible confounders such as hospital size or the presence of a TB screening program.

***Source:*** Prepared by the authors based on the study data.

#### Acknowledgements.

This study was possible thanks to the collaboration of the national and local tuberculosis program of the countries in which the survey was conducted.

#### Funding.

The study was funded by the United States Agency for International Development (Washington, DC, United States; Grant 002140). This institution did not have any role in study design, data collection, data analysis, or decision to publish.

#### Disclaimer.

Authors hold sole responsibility for the views expressed in the manuscript, which may not necessarily reflect the opinion or policy of the *RPSP/PAJPH* and/or PAHO.
